# Ten Prominent Host Proteases in Plant-Pathogen Interactions

**DOI:** 10.3390/ijms19020639

**Published:** 2018-02-24

**Authors:** Emma L. Thomas, Renier A. L. van der Hoorn

**Affiliations:** The Plant Chemetics Laboratory, Department of Plant Sciences, University of Oxford, Oxford OX1 3RB, UK; emma.thomas@biodtp.ox.ac.uk

**Keywords:** protease, plant, pathogen, defence, substrate, immunity, hypersensitive response, recognition, signalling, priming

## Abstract

Proteases are enzymes integral to the plant immune system. Multiple aspects of defence are regulated by proteases, including the hypersensitive response, pathogen recognition, priming and peptide hormone release. These processes are regulated by unrelated proteases residing at different subcellular locations. In this review, we discuss 10 prominent plant proteases contributing to the plant immune system, highlighting the diversity of roles they perform in plant defence.

## 1. Introduction

Proteases are ubiquitous and essential enzymes for life. Peptide bonds in proteins are hydrolysed by proteases, releasing peptides or amino acids. Proteolytic cleavage is thus an irreversible post-translational modification that has potent effects on protein behaviour. Proteases can degrade proteins, thereby terminating its function and removing it from the cell. Degradation also serves to recycle amino acids and enables reallocation of nitrogen resources [1]. Alternatively, protein cleavage can have a positive regulatory effect on proteins [2]. Proteases can mature substrate proteins through the removal of regulatory or inhibitory domains and, consequently, activate the catalytic or signalling activity.

Protease classification is dependent on the catalytic mechanism and is described in the MEROPS database [3]. The most prominent plant protease classes are cysteine, serine, threonine and aspartic proteases (named after their respective key catalytic residues) and metalloproteases [4]. The specificity of these proteases is dictated by the substrate amino acid sequence and 3D structure of the substrate.

Numerous biological processes in plants require proteases, including the plant defence response [4,5,6,7]. Plants can perceive pathogens by recognition of conserved pathogen-associated molecular patterns (PAMPs), such as flagellin or chitin, via membrane-localised pattern recognition receptors (PRRs). Alternatively, pathogen effectors are recognised, predominantly, by nucleotide-binding site leucine-rich repeat (NBS-LRR) proteins [8]. Following pathogen recognition, signalling events transduce crucial information on pathogen attack and coordinate intracellular and tissue-wide responses [9,10]. The cell undergoes large-scale transcriptional reprogramming and changes in salicylic acid (SA), jasmonic acid, and ethylene hormonal signalling to control local and systemic defence responses [11,12,13,14,15]. A common feature of defence is the hypersensitive response (HR), a form of programmed cell death occurring locally at the primary infection site. HR and local immune responses limit the spread of the pathogen and restrict their access to nutrients [16].

**Figure 1:**
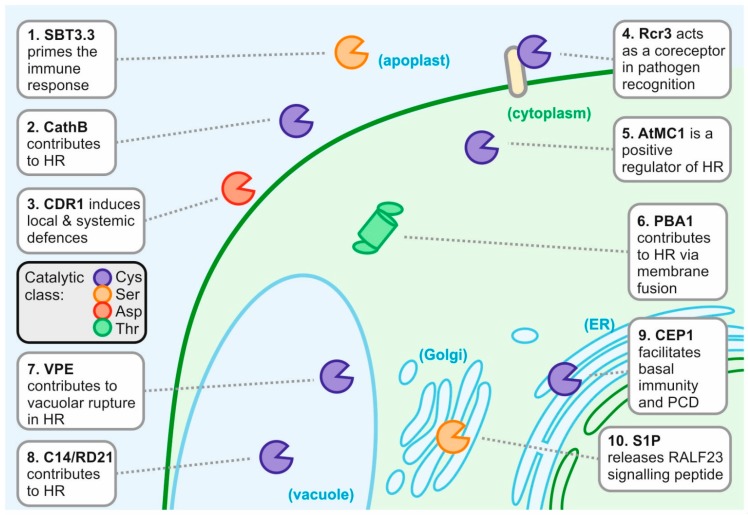
Ten host proteases contributing to the plant defence response. AtMC1, *Arabidopsis thaliana* metacaspase-1; C14, cysteine protease clone 14; CathB, cathepsin B; CDR1, constitutive disease resistance-1; CEP1, cysteine endopeptidase-1; HR, hypersensitive response; PBA1, proteasome beta subunit-1; PCD, programmed cell death; Rcr3, required for cladosporium resistance-3; RD21, responsive to dessication-21; S1P, site-1-protease; SBT3.3, subtilase 3.3; VPE, vacuolar-processing enzyme.

Increasing numbers of proteases have been implicated in different aspects of plant immunity. This review focuses on the roles of host proteases in plant-pathogen interactions discussed by their subcellular localisation (Figure 1). We highlight 10 examples of proteases with diverse roles in defence (Table 1) to illustrate both the different roles protease play in defence, and the diversity of proteases in the plant immune system.

## 2. Apoplastic Proteases

Early interactions between plant and pathogen occur in the apoplast. Many bacterial, fungal and oomycete pathogens start host colonisation in the apoplast, employing secreted effector molecules. Host proteins in the apoplast or plasma membrane play a role in the perception of pathogen-effector molecules and in extracellular defence signalling.

### 2.1. Subtilase 3.3 (SBT3.3) Regulates the Priming of the Plant Immune Response

Priming is the memory of a stress that enables the plant to launch an amplified and more rapid defence response upon future challenge. The *Arabidopsis* serine protease SBT3.3 (a subtilase member of the S8 family) regulates defence priming. *Arabidopsis sbt3.3* mutants are hypersusceptible to both the model bacterial pathogen *Pseudomonas syringae* and the oomycete *Hyaloperonospora arabidopsidis* [17]. Mutant *sbt3.3 Arabidopsis* plants are impaired in priming of both gene expression and signalling activity. Induction of mitogen-associated protein kinase (MPK) activity is enhanced upon SBT3.3 overexpression. Furthermore, SBT3.3 overexpression increases the abundance of transcriptional activating epigenetic marks at SA-regulated genes, including the promoters of WRKY transcription factors and SBT3.3, creating a positive feedback loop. Consequently, these genes are induced more rapidly upon pathogen challenge [17]. Interestingly, in addition to defence priming, SBT3.3 is required for SA-regulated gene activation. The mechanism of SBT3.3 controlling SA-regulated gene expression and priming the immune response remains enigmatic, as do the substrates of SBT3.3.

### 2.2. Cathepsin B (CathB) Is a Positive Regulator of Hypersensitive Response (HR)

The papain-like cysteine protease Cathepsin B (CathB, a C1 family member) is a positive regulator of HR. Multiple *CathB* genes contribute redundantly to basal resistance in *Arabidopsis* [18]. In *Nicotiana benthamiana*, CathB is secreted into the plant apoplast and activated [19]. Deficiency of CathB in *N. benthamiana* restricts programmed cell death (PCD) triggered by the bacterial pathogens *Erwinia amylovora* and avirulent *P. syringae*. Furthermore, PCD triggered by hydrogen peroxide, a prominent plant defence signal, and endoplasmic reticulum (ER) stress induced by tunicamycin requires CathB activity [20,21].

Whilst the involvement of CathB in HR is well established, the extent of its involvement depends on the HR inducer. CathB is required for HR triggered upon coexpression of *P. infestans* avirulence gene *AvrR3a* and potato resistance gene *R3a*, and upon *Ps* pv. *glycinea AvrB* expression [18,19]. However, CathB deficiency does not perturb HR upon co-expression of the *Cladosporium fulvum* avirulence gene *Avr4* and the tomato resistance gene *Cf-4* in *N. benthamiana* [18,19]. *Arabidopsis* CathB is also not necessary for resistance to *P. syringae* harbouring avirulence genes *AvrB* or *AvrRps4* [18]. The different roles indicate that CathB is important in multiple forms of HR, but is not necessarily a universal HR regulator.

### 2.3. Constitutive Disease Resistance-1 (CDR1) Promotes the Release of Systemic Defence Signals

CDR1 is an apoplastic aspartic protease of the A1 family that contributes to local and systemic defence signalling in *Arabidopsis*. Activation tagging of CDR1 results in enhanced resistance to multiple *P. syringae* strains, alongside constitutive pathogenesis related (PR) gene expression in a SA-dependent manner. PR gene expression is abolished in CDR1 active site mutants and upon application of the aspartic protease inhibitor pepstatin A, demonstrating that protease activity is required for the role in defence [22]. CDR1 generates an extracellular mobile signal capable of inducing defence responses both locally and systemically. Low molecular weight fractions of apoplastic fluids from CDR1 overexpressing plants induce defence responses in unchallenged plants, in both the infiltrated and distant leaves [22]. The activity of CDR1 appears to be conserved between species. Rice (*Oryza sativa*) CDR1 (OsCDR1) expressed in *Arabidopsis* similarly generates apoplastic fluids that induce systemic defence [23]. OsCDR1 overexpression in *Arabidopsis* also mimics the enhanced resistance to *Pst* observed on AtCDR1 overexpression [23]. The nature of the signal generated by CDR1 is currently unknown. Identification of the substrates of CDR1 will lead to insights into the systemic induction of SA-dependent defence responses.

### 2.4. Cysteine Protease Rcr3 Is a Coreceptor for Perception of Unrelated Pathogens

Recognition of a pathogen is the first step in mounting an immune response. The extracellular cysteine protease Rcr3 (family C1A) is crucial for the recognition of unrelated pathogens, including the fungus *C. fulvum* and nematode *Globodera rostochiensis*. Both the fungus and nematode secrete unrelated protease inhibitors (Avr2 and GrVAP1, respectively) that inhibit Rcr3 [24,25]. The inhibitor-Rcr3 complex is perceived by the tomato leucine-rich repeat receptor-like protein, Cf-2. Recognition triggers an oxidative burst, followed by transcriptional reprogramming and HR, culminating in disease resistance [25]. This is dependent on the presence of both Cf-2 and Rcr3. Rcr3 is proposed to act as a decoy with the operative effector target, Pip1, a paralogous and more abundant immune protease [26,27]. Deficiency of Pip1 renders the plant hyper-susceptible to *P. infestans*, *C. fulvum* and *P. syringae* [27]. In addition to the role in pathogen recognition, Rcr3 contributes to resistance via alternative pathways independent of *Cf-2*. *P. infestans* produces inhibitors of Rcr3 (EpiCs), but unlike *C. fulvum* and *G. rostochiensis* infection, these do not trigger HR. In the absence of Cf-2, *rcr3* mutants are hypersusceptible to *P. infestans*, but not to *C. fulvum* [27], indicating a Rcr3 role separate from Cf-2-dependent pathogen recognition.

## 3. Cytonuclear Proteases

The cytoplasm is an important signalling location that bridges the extracellular perception of pathogens and the intracellular responses, including changes in gene regulation, metabolite biosynthesis and induction of PCD. Cytoplasmic proteases have been implicated in HR regulation.

### 3.1. Arabidopsis thaliana Metacaspase-1 (AtMC1) Is a Positive Regulator of HR

Two cytosolic metacaspases, AtMC1 and AtMC2 (family C14), act antagonistically in the regulation of HR in *Arabidopsis*. AtMC1 positively regulates HR cell death induced by *P. syringae* carrying avrRPM1, although this HR does not affect pathogen growth [28]. Furthermore, AtMC1 is essential for the runaway cell death phenotype of defective immune components, including autoactive NLRs (Nod-like receptors, key *R* genes) and *lsd1* [28,29]. Consistent with its pro-cell death function, AtMC1 activity is tightly controlled by two negative regulators; LSD1 and AtSERPIN1 [28,30]. LSD1 directly interacts with AtMC1 through the N-terminal zinc finger domain [31], whereas suicide protease inhibitor AtSERPIN1 covalently and irreversibly inhibits AtMC1 [30]. The pro-death activity of AtMC1 is also suppressed by AtMC2. Overexpression of AtMC2 phenocopies the suppressed HR phenotype of *atmc1* mutants. Interestingly, whilst the role of AtMC1 in immunity requires its catalytic residues, the role of AtMC2 does not [28]. It is unknown how AtMC2 exerts the negative regulation of death independent of its protease activity, nor whether its catalytic activity contributes to alternative pathways in defence.

### 3.2. The Proteasome Is a Positive Regulator of HR

The host plant proteasome is essential for protein homeostasis and is heavily implicated in plant defence [32]. A notable example is that of NPR1, a transcriptional coactivator essential for SA-regulated gene expression. In non-induced cells, inappropriate transcription is restricted through degradation of NPR1 by the proteasome, whereas on SA induction, degradation is required for full transcriptional activation [33,34]. Degradation is proposed to increase the NPR1 recycling rate, thereby enabling greater gene expression.

The core particle of the proteasome is comprised of multiple subunits forming heptameric rings and include three catalytic β subunits with distinct proteolytic activities [35,36,37]. One catalytic subunit in particular, the threonine protease PBA1/β1 (of the T1 family), has been further investigated in the context of HR, due to its caspase-3-like activity. The presence of casapse-3-like activity is an established requirement for plant PCD, and in certain forms of PCD 60% of the caspase-3 like activity can be attributed to PBA1 [20,38,39,40]. Tobacco *PBA1* expression is induced following treatment with the fungal elicitor, cryptogein [41,42]. Deficiency of the PBA1 subunit compromises HR triggered by avirulent *P. syringae* carrying *AvrRpm1*. This HR is associated with the fusion of tonoplast and plasma membranes [38]. *PBA1*-silenced plants exhibit reduced activity of the other two catalytic subunits, PBB and PBE. Silencing these subunits replicates the suppression of HR, indicating a general role of the proteasome in HR induction [38]. PBA1-dependent HR is distinct from the regulation of gene expression, as induction of NADPH oxidases and PR genes are not suppressed in PBA1-deficient plants [38].

## 4. Vacuolar Proteases

The vacuole is an acidic hydrolytic storage compartment occupying the largest volume of a leaf cell. Rupture of the vacuole during HR dramatically alters the cytoplasm by acidification and the release of lytic enzymes and potential cell-death mediators [43,44,45]. Two vacuolar proteases have been identified that contribute to HR.

### 4.1. Vacuolar Processing Enzymes (VPEs) Regulate Vacuolar Rupture during Virus-Induced HR

Vacuolar processing enzymes (VPEs/Asparaginyl endopeptidases/Legumains, family C13) are key regulators of tonoplast integrity in PCD. VPEs cleave after asparagine (N) but can also cleave after aspartic acid (D) and, therefore, have caspase-1-like activity. VPEs are essential for vacuolar rupture and HR upon infection by tobacco mosaic virus (TMV) on *N. benthamiana* carrying the N resistance gene [46]. Similarly, upon ER stress-induced PCD, absence of VPEs prevents vacuolar rupture. VPEs mature autocatalytically and are known to activate another protease, AtCPY, in the vacuole [43,47]. VPEs may, therefore, be key regulators of the PCD-induction pathway.

Despite the lack of known substrates of VPEs during PCD, the requirement of VPEs for HR is well described [48,49]. VPEs are also required for HR triggered by mycotoxin FB1, bacterial elicitor harpin, and the co-expression of calcium channels CNGC11 and CNGC12 [46,50,51,52]. Nonetheless, the role of VPEs is not universal. HR induced by boehmerin and Nep1 is not perturbed upon VPE silencing [51].

VPE-mediated tonoplast rupture is thought to be effective against cytoplasmic pathogens like viruses that become exposed to vacuolar hydrolases and low pH [53]. In contrast, fusion of the tonoplast and plasma membranes is dependent on PBA1 and delivers vacuolar contents to the apoplast where bacteria reside [38]. Crucially, however, these morphologies were observed in different plant species, *N. benthamiana* and *Arabidopsis*, respectively. To date, it is unclear how widespread the different forms of HR PCD are.

### 4.2. Papain-Like Proteases C14/RD21 Have a Complex Regulation

C14 and RD21 are orthologous papain-like proteases from tomato and *Arabidopsis*, respectively [54], carrying a C-terminal granulin domain [55]. Tomato C14 has been detected in the vacuole [56] and extracellularly [26] C14 probably plays an important role in immunity because its activity and localisation are manipulated by multiple effectors. The extracellular C14 is targeted by cystatin-like EpiC inhibitors of the oomycete pathogen *P. infestans* [57], and the chagasin-like Cip1 inhibitor of the *P. syringae* [58]. In addition, RxLR effector AvrBlb2 of *P. infestans* associates with C14 and prevents its secretion into the apoplast [56].

Importantly, silencing or overexpression of a C14 homolog in *N. benthamiana* enhances or decreases susceptibility to *P. infestans*, respectively [56,57]. However, *Arabidopsis rd21* knock-out lines are not more susceptible to the oomycete *H. arabidopsidis*, even though they express genes encoding EpiC-like inhibitors [54]. Nevertheless, these *rd21* lines are more susceptible to *Botrytis cinereal* when whole plants are infected [54]. Remarkably, the opposite phenotype with *B. cinereal* (increased resistance) was found for the same *rd21* mutants in detached-leaf assays [59]. These data indicate that the role of C14/RD21 proteases depends on the pathosystem, the assay itself and on the different ways pathogens manipulate their host.

Control over RD21 activity upon release of the vacuolar content into the cytoplasm during PCD is thought to come from AtSERPIN1, a cytoplasmic serpin-like suicide inhibitor that forms a covalent complex with RD21 [59]. Indeed, AtSERPIN1 overexpression causes susceptibility to *B. cinereal* [59]. However, *atserpin1* mutants do not show a phenotype and AtSERPIN1 also regulates PCD via AtMC1 [30]. Besides AtSERPIN1, RD21 is also regulated by kunitz inhibitor WSCP [60], protein di-isomerase PDI5 [61], and other mechanisms [62]. This makes RD21 regulation a challenging and intriguing question to resolve.

## 5. Endomembrane Proteases

The endomembrane system includes the endoplasmic reticulum and the Golgi network which are important for protein synthesis and maturation. Stress responses rely heavily on protein production to enable the cell to adapt [63]. Endomembrane compartments are also involved on many viral, fungal and oomycete infections—for instance, by flanking pathogen haustoria—and have been implicated in PCD initiation [64].

### 5.1. Endoplasmic Reticulum (ER) Resident AtCEP1 Facilitates Fungal Immunity

AtCEP1 is a specific, papain-like cysteine endopeptidase (family C1A) that harbours a C-terminal ‘KDEL’ sequence that sequesters the protease within ER-derived compartments. The expression of AtCEP1 is induced upon infection with the fungal obligate biotroph *Erysiphe cruciferarum* where it contributes to basal resistance [65,66]. Expression of green fluorescent protein (GFP) fusion constructs revealed AtCEP1 is enriched in endomembranes surrounding the haustorium interface during HR induction [66]. However, AtCEP1 contains a putative cleavage site that would result in the loss of the KDEL sequence, and therefore AtCEP1 activity may also be present elsewhere [65].

*Arabidopsis atcep1* mutants are hypersusceptible to *E. cruciferarum* [65,66]. Cells penetrated by fungal haustoria characteristically undergo PCD and this is reduced in *atcep1* mutants [65,66]. AtCEP1 is also implicated in developmental forms of PCD, specifically tapetal PCD [67]. Expression of AtCEP1 is under regulation by CPR5, a major regulator of PR gene expression [66,68]. PCD on *E. cruciferarum* infection is also controlled by CPR5 [66]. Conversely, however, *cpr5* mutants are resistant to *E. cruciferarum* and exhibit spontaneous cell death, in a manner epistatic to AtCEP1 [66]. The deregulation of AtCEP1 in *cpr5* mutants is thought to contribute to the excessive cell-death phenotype.

### 5.2. Golgi-Localised Site-1-Protease (S1P) Controls Rapid Alkalinisation Factor 23 (RALF23) Peptide Signalling

The Golgi-localised subtilase Site-1-Protease (S1P/SBT6.1, family S8) presents a rare example of a protease in immunity with not only a verified substrate but also a known role for its identified substrate. S1P processes rapid alkalinisation factor 23 (RALF23) into a mature signalling peptide [69,70]. RALF23 is perceived extracellularly by the transmembrane malectin-like receptor kinase FERONIA [71]. Perception of RALF23 dampens immune signalling through the inhibition of PRR complex formation, thus restricting excessive defence responses that may prove costly to the plant [71]. S1P is, therefore, an important intracellular subtilase that negatively regulates the immune response.

Regulation of S1P could be a mechanism to rapidly control the abundance of mature RALF23 and, thereby, fine-tune the immune response. Indeed, both S1P activity and RALF23 abundance rapidly increase upon challenge with *P. syringae*. RALF23 is an important substrate of S1P, as both *s1p* and *ralf23* plants exhibit comparable enhanced reactive oxygen species (ROS) bursts and resistance to *P. syrignae* [71]. Remarkably, RALF peptide mimics have also been identified in pathogenic fungi and are contributors to virulence [72], indicating that this signalling pathway may be a core component of immunity in plants.

## 6. Discussion

Proteases have diverse roles in the plant immune system, ranging from pathogen perception (Rcr3), defence priming (SBT3.3), signalling (CDR1 and S1P) to regulation of HR (CathB, AtMC1, PBA1, VPEs, RD21 and AtCEP1). Whilst we have highlighted just 10 prominent examples here, this review is not comprehensive and many more host proteases are involved in plant-pathogen interactions. 

The large number of proteases involved in HR is to be expected considering the importance of proteases in animal PCD. Cysteine proteases known as caspases are essential for animal PCD in disease, acting as both regulators and executioners of cell death [73,74,75]. The absolute requirement for caspases has led to a longstanding bias in plant research that proteases with caspase-like activities are important in plant PCD. While this is true for VPEs, CathB and PBA1/proteasome, proteases without caspase-like activity are also important in HR. Furthermore, unlike caspases, plant proteases involved in HR are of unrelated families. CathB (C1A), AtMC1 (C14), RD21 (C1A), PBA1 (T1) and VPEs (C13) are all implicated in HR regulation, and represent diverse protease classes [18,38,48,59,76].

Interestingly, evidence from studies on proteases involved in HR demonstrate that HR can be genetically uncoupled from the restriction of pathogen growth. Of the proteases discussed, CathB and AtMC1 both contribute to HR, independent of restricting pathogen growth [18,28]. Furthermore, the inconsistent requirements for the CathB, VPEs, PBA1 and RD21 proteases in HR indicate that multiple pathways to HR are present. Parallel pathways to HR would be advantageous to avoid essential nodes in immune-defence networks that may be targeted by effectors and increase susceptibility to a broad range of pathogens.

Strikingly, there are no examples yet of proteases directly degrading pathogen proteins. There are a number of proteases linked to defence whose role in immunity is completely unknown. For example, P69B is frequently implicated in pathogen defence, but precisely what role it plays is not clear [24,27,77,78,79,80]. Moreover, other proteases that already have an identified role may possess additional functions in defence.

The requirement for host proteases in plant-pathogen interactions is clear, but the mechanism in which they act is frequently not. The major factor limiting our understanding of protease roles is the general lack of known, biologically relevant, substrates. Without this knowledge, it is impossible to fully understand the mechanism of a protease in immunity. Of the proteases discussed in this review, a biologically relevant substrate has only been identified for S1P [69]. Although the evidence is compelling for RALF23 being the major substrate of S1P in the context of defence, due to RALF23 depletion and its overexpression phenocopying that of S1P [71], it is highly unlikely that proteases have exclusively one substrate. Furthermore, it is important to validate whether protease mutant phenotypes result from loss of protease activities, by including catalytically dead mutants. For most immune proteases, this control has not been included. This leaves open the possibility that other protein functions may be contributing to immune phenotypes. 

Proteases do not act in isolation in immunity, and protease mutant phenotypes may be indirect. In humans, a computational study demonstrated that proteases impact activities of other proteases in a complex web [79]. This interconnectivity is compounded by multifunctional inhibitors [80] such as AtSERPIN1, which regulates several unrelated immune proteases [30,59,81]. In addition, many proteases can possess similar activities, such as CathB and PBA1 exhibiting caspase-3-like activity, which could therefore act redundantly. Deciphering the roles of individual proteases in defence is by no means a trivial task. 

It is important to note that subcellular localisations may be dynamic, especially upon stress. This is illustrated by CathB, which is not restricted to the apoplast. Mass spectrometry data from unchallenged *Arabidopsis* plants supports a vacuolar localisation, whilst expression of red fluorescent protein (RFP) fusions and apoplastic activity assays in *N. benthamiana* support CathB presence in the apoplast [18,82]. It may be possible that either the protease has a dual localisation, is relocated upon different stresses, or its localisation differs between species. The Golgi-localised S1P has also been detected in the apoplast, where it interacts with and is inhibited by AtSERPIN1 [82]. AtSERPIN1 has been identified in the cytoplasm, Golgi, ER and apoplast [59,83]. Thus, caution should be exercised when assuming the location in which proteases mediate their phenotype.

Our current knowledge of the exact roles of proteases places them as key players in many facets of pathogen responses. Future efforts in this field will need to address the lack of known substrates e.g., by applying novel mass-spectrometry based methods [84,85], the assignment of subcellular localisation, and the role of proteases in interactions with different pathogens. Despite these limitations, proteases are now well established as important contributors to host defence. Future research addressing their regulation and substrates will undoubtedly produce greater insights into the plant immune system.

## Figures and Tables

**Table 1:** Ten prominent host proteases in plant-pathogen interactions.

	Function in Defence	Subcellular Localisation	MEROPS Family	Organism	Known Substrate in Defence?
1. SBT3.3	Priming	Apoplast	S08, subtilisin-like	*A. thaliana*	No
2. CathB	Hypersensitive response (HR)	Apoplast (+Vacuole)	C01, papain-like	*N. benthamiana*, *A. thaliana*	No
3. CDR1	Signalling	Apoplast	A01, pepsin-like	*A. thaliana*	No
4. Rcr3	Recognition	Apoplast	C01, papain-like	Tomato	No
5. AtMC1	HR	Cytoplasm (+Nucleus)	C14, metacaspase	*A. thaliana*	No
6. PBA1	HR, membrane fusion	Cytoplasm	T01, proteasome	*A. thaliana*	No
7. VPE	HR, membrane fusion	Vacuole	C13, legumain-like	*N. benthamiana*, *A. thaliana*	No
8. C14/RD21	HR, resistance	Vacuole	C01, papain-like	Tomato, *A. thaliana*	No
9. CEP1	Basal resistance	Endoplasmic reticulum (ER) derived compartments	C01, papain-like	*A. thaliana*	No
10. S1P	Signalling, hormone release	Golgi	S08, subtilisin-like	*A. thaliana*	RALF23

MEROPS database [3]. The major subcellular localisation is named, locations in brackets are other reported localisations.
